# Robustness in Glyoxylate Bypass Regulation

**DOI:** 10.1371/journal.pcbi.1000297

**Published:** 2009-03-06

**Authors:** Guy Shinar, Joshua D. Rabinowitz, Uri Alon

**Affiliations:** 1 Departments of Molecular Cell Biology and Physics of Complex Systems, Weizmann Institute of Science, Rehovot, Israel; 2 Department of Chemistry and Lewis-Sigler Institute for Integrative Genomics, Princeton University, Princeton, New Jersey, United States of America; University of Virginia, United States of America

## Abstract

The glyoxylate bypass allows *Escherichia coli* to grow on carbon sources with only two carbons by bypassing the loss of carbons as CO_2_ in the tricarboxylic acid cycle. The flux toward this bypass is regulated by the phosphorylation of the enzyme isocitrate dehydrogenase (IDH) by a bifunctional kinase–phosphatase called IDHKP. In this system, IDH activity has been found to be remarkably robust with respect to wide variations in the total IDH protein concentration. Here, we examine possible mechanisms to explain this robustness. Explanations in which IDHKP works simultaneously as a first-order kinase and as a zero-order phosphatase with a single IDH binding site are found to be inconsistent with robustness. Instead, we suggest a robust mechanism where both substrates bind the bifunctional enzyme to form a ternary complex.

## Author Summary

To grow well, the cell needs to produce a balanced set of building blocks by means of its metabolic network. Regulatory circuits are used to maintain appropriate fluxes as metabolites flow through the branching pathways in the network. Here, we asked how such regulatory circuits can work precisely, despite the fact that they are made of proteins whose levels vary from cell to cell and in the same cell over time. We used a well-studied circuit, at a key branch point called the glyoxylate bypass, as a model system. Previous experiments showed that this system is remarkably robust to changes in the levels of its proteins. Here, we propose a mechanism to explain this robustness, based on a bifunctional enzyme that catalyzes two opposing reactions. We show that a simple explanation based on enzyme saturation is inconsistent with more rigorous mathematical analysis. Our proposed mechanism suggests several experimentally testable predictions. It shows how a systems-level feature (robustness) may arise from seemingly unrelated biochemical details. Because analogous designs with bifunctional enzymes are found in other systems in different organisms, the present mechanism might apply more broadly.

## Introduction

Robustness in biological systems has seen a renewal of research interest in recent years [1]–[12]. To define robustness, one needs to specify what feature is robust and with respect to which variations. Classic experimental studies have shown that metabolic fluxes are often insensitive to the levels of enzymes in the pathway, as reviewed in [13]. Metabolic control theory addresses this by suggesting that control of flux is distributed amongst many enzymes, and thus no single enzyme is rate limiting.

In the last decade, studies have added a new level of understanding on robustness by providing detailed molecular mechanisms that can preserve the essential function of a system in the face of large variations in the protein levels. For example, specific mechanisms explain how exact adaptation in bacterial chemotaxis is robust with respect to chemotaxis protein levels [2],[3], and how patterning in drosophila embryos is robust with respect to morphogen production rates [12],[14],[15]. A recent review summarizes experiments and theoretical mechanisms for robustness [10].

Recently, an intriguing class of robust mechanisms has been found, based on bifunctional enzymes that carry out two opposing reactions (such as both modifying a target protein, and removing the modification) [8],[11]. These robust mechanisms seem to apply to a class of bacterial two-component signaling system. These systems show robustness of input-output relations, in the sense that output responds to input signals in a way that is not disrupted by variations in protein levels.

Here, we extend this line of research to one of the best studied regulation steps in *E. coli* metabolism, the IDHKP/IDH system. This system raised our interest because it employs a bifunctional enzyme that carries out two opposing reactions, hinting at a robust mechanism. However, it has several biochemical differences from previously studied systems [8],[11], suggesting that it may show a new type of robust mechanism.

**Figure 1:**
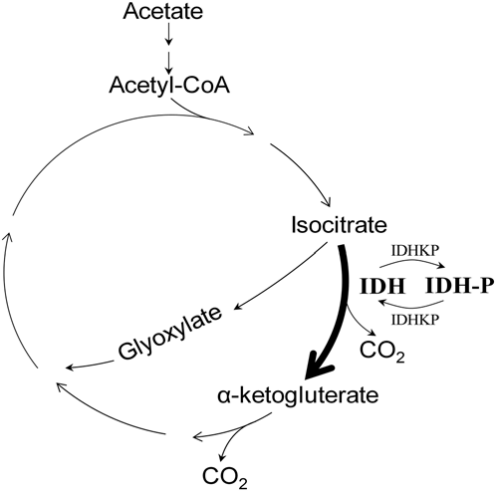
The glyoxylate bypass. IDH denotes the unphosphorylated and active form of isocitrate dehydrogenase. IDH-P denotes the phosphorylated and inactive form.

The need for precise regulation in the IDHKP/IDH system is evident from its biological function. The IDH system regulates the partitioning of carbon flux between the TCA cycle and the glyoxylate bypass (Figure 1). Precise regulation of flux to the glyoxylate bypass is essential when the bacterium grows on substances such as acetate that contain only two carbon atoms. Without the glyoxylate bypass, both carbon atoms would be converted to CO_2_ by the TCA cycle, thereby leaving no carbon available for biosynthesis of cell constituents. Hence, growth on acetate and other two-carbon compounds requires directing some of the carbon flux to the glyoxylate bypass, thereby avoiding carbon loss.

The precise partitioning of carbon flux between the cycle and the bypass is achieved by regulating the activity of the enzyme IDH (isocitrate dehydrogenase), which stands at the entry to the bypass. The activity of IDH is determined by its phosphorylation state: only unphosphorylated IDH is active. During growth on substances with more than two carbon atoms, IDH is mostly unphosphorylated and hence active. Thus, most of the carbon flux is directed to the more efficient TCA cycle. On the contrary, during growth on acetate, most of IDH is phosphorylated and hence inactive, so that a large part of the carbon flux is directed to the bypass [16]–[19].

To regulate the IDH phosphorylation level, *E. coli* employs a bifunctional enzyme. This enzyme catalyzes both the phosphorylation of IDH, and its dephosphorylation, and is called IDHKP (IDH Kinase/Phosphatase) [20]. IDHKP uses ATP as the phosphoryl donor for the kinase reaction, and also requires ATP as a cofactor for the dephosphorylation reaction [20]–[22]. The activity of IDHKP is allosterically regulated by the levels of various metabolites in the cell that act as the input signals to this system [21].

The robustness of IDH activity has been experimentally tested by Laporte et. al. [23]. It was found that during growth on acetate, the concentration of active (unphosphorylated) IDH is extremely robust: The level of active IDH changes by less than 20% upon 15-fold variation in total IDH concentration.

What is the mechanism for this robustness? It was suggested in [23] that the robustness of active IDH levels may result either from regulation of the activity of IDHKP by putative modulators sensitive to the metabolic state of the cell, or by a specific mechanism whereby *the enzyme IDHKP works simultaneously as a first-order kinase and as a zero-order phosphatase*. We quote from [23]:

“The second mechanism for maintaining a constant level of isocitrate dehydrogenase activity rests upon the inherent kinetic parameters of the modifying enzymes. During log phase growth on acetate, the kinase is operating essentially in the first order region and the phosphatase is saturated with substrate [18]. As a result, the velocity of the phosphatase is independent of substrate concentration over a wide range. Consequently, the steady-state concentration of the phosphatase's substrate will vary, but the substrate of the kinase, isocitrate dehydrogenase, will remain nearly constant.”

The simplest model corresponding to the argument above is as follows: 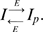
(1)
*I* denotes active IDH, *I_p_* denotes phosphorylated IDH, and *E* denotes the bifunctional enzyme IDHKP. The arrows in (1) *do not* denote full chemical reactions. Rather, they symbolize enzyme catalysis steps. The behavior of the model depends on the details of the actual chemical reactions involved. The simplest mass-action kinetic system corresponding to (1) is
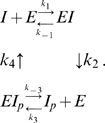
(2)This system assumes a single binding site (shared by *I* and *I_p_*) on the bifunctional enzyme *E*.

An intuitive analysis of (1) would involve assigning, in the usual way, Michaelis-Menten rate functions *f*_1_ and *f*_2_ to the “reactions” 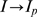 and 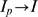, respectively:
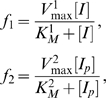
(3)where
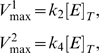
(4)

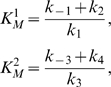
(5)and [*E*]*_T_* is the time-conserved total concentration of *E*:
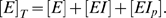
(6)Note that this analysis ignores the possibility of *I* and *I_p_* competing for the active site of *E*.

Subsequently, assume that the rate constants in (2) are such that *E* works as a first-order kinase and a zero-order phosphatase [18], [24]–[27]: that is, 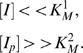
(7)Then, using (7) in (3) gives
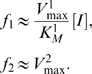
(8)


At steady-state, the rates of enzyme-catalyzed phosphorylation and dephosphorylation are equal:
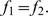
(9)Using (4), (5), and (8) in (9) yields
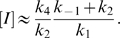
(10)Inspection of (10) shows that, under the assumptions made, [*I*] is insensitive to changes in [*I*]*_T_*. Thus, the robustness of [*I*] with respect to [*I*]*_T_* is apparently explained.

In the present study we show that the full mass-action kinetic model (2) cannot give rise to equation (10), regardless of the choice of parameters. In fact, we demonstrate that for all parameter choices, the ratio [*I*]/[*I_p_*], not [*I*], is robust at steady state. Thus, (2) cannot account for the experimentally observed robustness of [*I*]. From this it follows that the use of Michaelis-Menten rate functions (3) to derive (10) is inconsistent with the “parent” mass-action model (2), due to competition of *I* and *I_p_* for the active site of *E*.

We then propose mass-action models that explain how robustness might arise in the IDHKP/IDH system. A common feature of these models is the formation of a ternary complex between *I*, *I_p_*, and *E*.

## Results

### Mass-Action System (2) Does Not Give Rise to Robustness of [*I*]

Our goal, in this section, is to show that mass-action system (2) cannot give rise to equation (10), and to robustness. This, once demonstrated, implies that there is a flaw in the derivation of equation (10), namely, in the assumption that the Michaelis-Menten approximation without competition applies.

We begin by writing the differential equations corresponding to mass-action system (2).
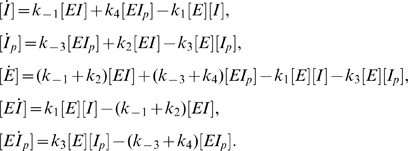
(11)Equations (11) are consistent with (6), as well as with the conservation of total *I*:
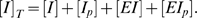
(12)From the second and fifth equations in (11) we have that
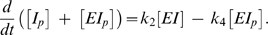
(13)Considering the last two equations of (11) and equation (13) at steady-state we obtain the ratio between the active and the inactive forms of *I*:
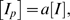
(14)where
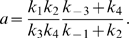
(15)


Equation (14) shows that system (2) implies that the ratio [*I*]/[*I_p_*] is robust: [*I*]/[*I_p_*] = *a* ^−1^. Robustness of [*I*]/[*I_p_*] obtains because it does not depend on protein levels, only on rate constants. *This happens regardless of the choice of parameters in the system.* Moreover, if we assume that enzyme *E* is rare compared to its substrate,
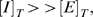
(16)we can approximate
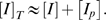
(17)Using (17) in (14) we find that the unphosphorylated (and thus active) form *I* depends on the total *I* level:
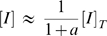
(18)In other words, not only system (2) fails to show robustness of *I* activity in the face of variations in the total *I* protein level, but also the dependence of [*I*] on [*I*]*_T_* at steady-state is linear.

More generally, the inconsistency between (10) and (18) implies that the Michaelis-Menten approximation, which applies to each phosphorylation and dephosphorylation reaction alone, cannot apply when both reactions are simultaneously catalyzed by the same bifunctional enzyme with a single site for which *I* and *I_p_* compete.

#### Remark

Inequality (16) is reminiscent of the criterion 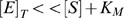 which guarantees the validity of the quasi steady-state assumption for the simple Michaelis-Menten reaction 
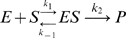
. [Here 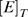 is the time-conserved quantity 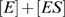, and 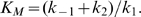]. In system (2), however, the quasi steady-state assumption is not required for deriving (18), and therefore, (16) is not employed to insure quasi steady-state. Rather, (16) is used to guarantee the approximate conservation law (17), which is necessary for arriving at (18). The quasi steady-state approximation, and the more general total quasi steady-state approximation are described in [28],[29].

### A Putative Mass-Action Model That Explains the Robustness of [*I*]

Our aim, in this section, is to construct a mass-action model that can explain how a high degree of robustness of [*I*] with respect to variations in [*I*]*_T_* can be achieved.

Following Goldbeter and Koshland [26], we will view phosphorylation and dephosphorylation as irreversible modifications, and will not explicitly account for ATP, ADP and phosphoryl ions. This allows a clear understanding of the model.

The model begins with the bifunctional enzyme *E* that phosphorylates *I* and dephosphorylates *I_p_*, as described in the reactions in (2). To obtain robustness requires several additional assumptions. Most importantly, we need to suppose that *E* has two distinct binding sites: one for *I* and one for *I_p_*. This is suggested by the fact that mutant *E. coli* strains have been isolated where *E* has greatly reduced phosphatase activity but retains the kinase activity [22]. Kinetic studies on these mutants show that they have a 40-fold reduction in their affinity to *I_p_*, whereas their affinity to *I* remains virtually the same as in the wild-type [22].

In addition, we assume that the ternary complex *EI_p_I* can form and has kinase activity. In our initial analysis, we shall also assume that the ternary complex *EI_p_I* has *only* kinase activity, and that the ternary complex forms in an *ordered* fashion, that is, first *E* binds *I_p_* and then *EI_p_* binds *I* (both assumptions will later be relaxed.) Thus, we propose the following mass-action model:
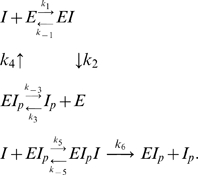
(19)


The differential equations corresponding to the mass-action reactions of (19) are
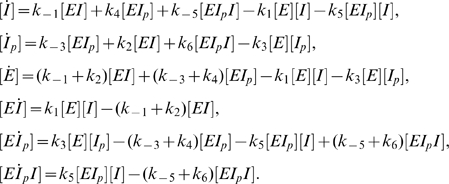
(20)


Summing equations in (20) shows conservation over time of the total *I* protein level,
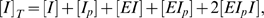
(21)and total E protein level,
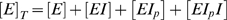
(22)As before, we will consider the physiologically relevant case where the substrate *I* is much more abundant than the enzyme *E* and thus
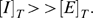
(23)Using (21) and (23) we see that equation (17) is valid in the present case. (Note that here, as in the case of system (2), the use of (23) is required for deriving the approximate conservation law (17), and not for ensuring quasi steady-state.)

By summing the second, fifth and sixth equations in (20) we find that
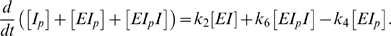
(24)


We now consider the last three equations in (20) and equation (24) at steady state. This gives a balance of phosphorylation and dephosphorylation rates,
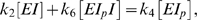
(25)and a set of relations between the concentrations of complexes and the product of the concentrations of their constituent elements:
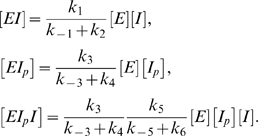
(26)


Using (26) and (17) in (25) we obtain a quadratic equation for the steady-state value of [*I*]:
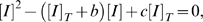
(27)where the parameters *b* and *c* are functions of the rate constants of the system:
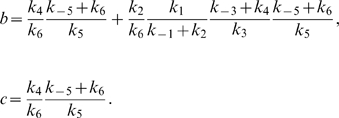
(28)Note for later use that
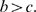
(29)From (27) and (29) we see that there is a unique solution for the steady-state value of [*I*], which satisfies the requirement 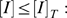


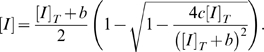
(30)


Inspection of (30) shows that [*I*] is robust with respect to changes in [*E*]*_T_*_ ,_ because [*E*]*_T_* does not appear in the equation for [*I*]. In general, [*I*] depends on [*I*]*_T_*, but robustness results when
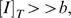
(31)which implies, by (29), that
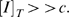
(32)As a result one obtains from neglecting *b* with respect to [*I*]*_T_* in (30), and then Taylor-expanding the resulting expression with respect to the small parameter 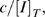 That
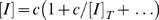
(33)This shows that for large values of [*I*]*_T_* compared to *b* and *c*, [*I*] (and thus *I* activity) is highly robust with respect to variations in the total *I* level.

### Ordered Binding and Kinase-Only Activity of the Ternary Complex Are Not Essential for Robustness

What happens if we relax the assumptions that the ternary complex can form only in the ordered fashion of (19) and has only kinase activity? We then need to consider the mass-action system
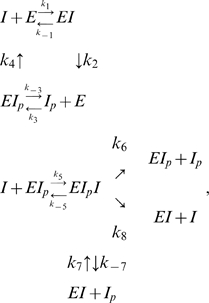
(34)which gives rise to the ordinary differential equations
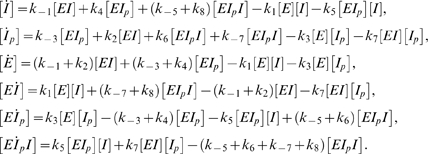
(35)Here, an analytic expression for [*I*] as a function of [*I*]*_T_* is no longer obvious, even if (23) is used. Nevertheless, in cases where (23) and (31) apply, and the ternary complex has stronger kinase than phosphatase activity 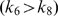, numerical analysis of (35) suggests that the steady-state value of [*I*] is approximately robust over a large range of [*I*]*_T_* values (see Methods). Moreover, if the ternary complex has much more kinase activity than phosphatase activity 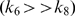, we have that the steady-state value of [*I*] is well approximated by the leading term in (33):
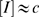
(36)(see Methods). Thus, even if the assumptions that the ternary complex must form in the ordered fashion of (19) and that the ternary complex has only kinase activity are relaxed, approximate robustness occurs over a large range of [*I*]*_T_* values.

We note that if we maintain the assumption of ordered binding, but now with *E* binding *I* first and then *EI* binding *I_p_* to form *EI_p_I*, simulations suggest that, subject to (23), (31) and 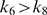, robustness of [*I*] with respect to [*I*]*_T_* is lost, and in fact 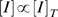 (see Methods).

Finally, we observe that (34) is symmetric with respect to exchanging the index “*p*.” Thus, if *I* is exchanged with *I_p_*, *EI* is exchanged with *EI_p_*, *EII_p_* is identified with *EI_p_I*, and the rate constants are suitably relabeled, then (34) remains invariant. This implies that if the ternary complex has more phosphatase than kinase activity then *I_p_* becomes the approximately robust species.

### Intuitive Explanation for the Robustness in the Proposed Mechanism

Let us intuitively understand the origin of robustness in (19). When [*I*]*_T_* is sufficiently large, that is, when 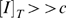, most of *I* is phosphorylated and found in the form *I_p_*. Hence, *E* is saturated with *I_p_*. This implies that most of the kinase activity is carried out by the abundant ternary complex *EI_p_I*, whereas the phosphatase activity is carried out only by the binary complex *EI_p_*. This situation can be approximately described by the mass-action system
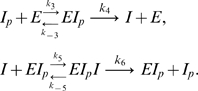
(37)Because at steady-state the rates of phosphorylation and dephosphorylation are equal, and because these rates are proportional to [*EI_p_I*] and [*EI_p_*], respectively, it follows that at steady state
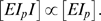
(38)Because *E* is saturated with *I_p_* and *EI* is neglected, the ternary complex is effectively formed in an ordered fashion, with *I_p_* binding first and *I* binding second. This, through the second equation in (37), constrains the equilibrium concentration of *EI_p_I* to be proportional to the product of the concentrations of its constituent species: that is,
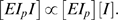
(39)Using (39) in (38) gives
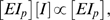
(40)which, following the cancellation of [*EI_p_*] from both sides of (40), yields the robust result
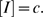
(41)This shows that [*I*] is independent of the total level of both proteins in the system ([*I*]*_T_* and [*E*]*_T_*). The “cancellation principle” used above [11] is related to that which demonstrates how robustness [8],[11],[30],[31] may arise in the EnvZ/OmpR system of *E. coli*.

It is important to note that the activity of *I* is still a function of the allosteric inputs to the enzyme *E*, through the rate constants that determine the parameter *c*. One may say that equation (41) describes a robust input-output relation [11] between IDH activity (output) and the allosteric effectors of IDHKP (inputs). This input-output relation is not affected by fluctuations in the total levels of the enzymes in the system.

## Discussion

This study suggests a new mechanism for the robustness in the glyoxylate bypass regulation of *E. coli*. The experimentally observed robustness of IDH activity in this system does not necessarily follow from intuitive arguments about first- and zero-order kinetics of the bifunctional regulator IDHKP. Rather, robustness requires specific biochemical features. These features work together to allow IDH activity to be highly insensitive to variations in the levels of the proteins in the system. While IDH activity is robust to protein levels, it is still responsive to input signals that affect rate constants. Thus the system may be said to have a robust input-output relation [11], where IDH levels respond to input signals in a reliable way that is not disrupted by fluctuation in enzyme levels.

The present mechanism for robustness relies, in addition to the known features of the system, on the assumption that the ternary complex *EI_p_I* exists. In addition, robustness in the present model requires that the ternary complex has more kinase than phosphatase activity. This role for a ternary complex in robustness adds to previous observations that relate ternary complexes to robustness and bistability [11],[32],[33].

The present model may explain a seemingly paradoxical aspect of the system. This effect occurs when *E. coli* is shifted from glycerol to acetate (where robustness has been observed). Despite the fact that IDH activity decreases in acetate compared to glycerol, the total IDH protein level *increases* due to upregulated gene expression [19]. The present model may explain this puzzle by showing that robustness under acetate conditions requires that [*I*]*_T_* levels are sufficiently high.

The present model is quite general: it may apply to other systems with a bifunctional enzyme that catalyzes antagonistic reactions. A possible example is the pyruvate, ortho-phosphate dikinase (PPDK) enzyme of plants [34].

The details of the proposed mechanism can be tested experimentally. To test for the existence of the ternary complex, one may construct two tagged versions of IDH, each with a *different* tag, and test if they co-immunoprecipitate *only* in the presence of the bifunctional enzyme IDHKP and ATP. Another experiment involves labeling in-vitro preparations of *I* with CFP (cyan fluorescent protein) and *I_p_* with YFP (yellow fluorescent protein), and adding saturating amounts of both to IDHKP and ATP. In the proposed mechanism, this should result in FRET (fluorescent resonance energy transfer) via the ternary complex.

If the ternary complex is shown to exist, then the next step is to test whether it has more kinase than phosphatase activity. One possible way to do this is to prepare *CFP-I* and *YFP-I_p_* where the phosphate is radioactive. One then adds saturating amounts of *CFP-I* and *YFP-I_p_* to IDHKP and ATP where the γ-phosphate is radioactive. Then, we expect that *CFP-I_p_* would form faster than *YFP-I*. This could be checked by immunoprecipitating and measuring the immunoprecipitates for radioactivity and color at several time points.

Finally, the current robust model was derived using mass-action kinetics and not Michaelis-Menten approximations. For bifunctional enzymes, care must be taken to explicitly consider competitive and cooperative effects before applying Michaelis-Menten approximations, as standard Michaelis-Menten behavior will not necessarily arise from “parent” mass-action systems. Further research can aim to specify conditions where Michaelis-Menten approximations are applicable, and to define the general classes of systems that can show robust properties [35].

## Methods

We studied mass-action system (34) using numerical simulations. We considered three scenarios: (a) The ternary complex forms in a random order. (b) The ternary complex forms in an ordered fashion, with *E* binding *I_p_* first and then *EI_p_* binding *I*. (c) The ternary complex forms in an ordered fashion, but now with *E* binding *I* first and then *EI* binding *I_p_*. All simulations were performed using Matlab. In each iteration, we studied (a), (b) and (c) in the following way: First, we chose each rate constant (with the exception of *k*_8_) in mass-action system (34) from a lognormal distribution with (natural) log mean equal to 0 and (natural) log standard deviation equal to 1. To ensure that 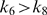, we chose *k*_8_ randomly from the interval [0.1*k*_6_, 0.9*k*_6_]. The parameters *b* and *c* were calculated according to (28). To ensure that (23) is met, the conserved total enzyme concentration [*E*]*_T_* was chosen randomly from the interval [0.1*c*,*c*], and [*I*]*_T_* was assigned the values *r*_1_ = 1000*b*, 1100*b*,…,2000*b* = *r*_2_. For each value of [*I*]*_T_*, we chose the initial conditions in the standard way, with *E*(0) = [*E*]*_T_*, *I*(0) = [*I*]*_T_*, and the initial concentrations of the remaining chemical species set to 0. The differential equations (35), which correspond to scenario (a) of random binding, were then integrated for each value of [*I*]*_T_* using the “ode23s” differential equation solver. The corresponding steady-state values of [*I*] were extracted. To analyze case (b), we repeated the exact same procedures as in (a), but with *k*_7_ and *k*_−7_ set equal to 0. Similarly, to analyze case (c), we repeated the procedures in (a), but now with *k*_5_ and *k*_-5_ set equal to 0. We performed a total of 10,000 simulation runs for each of the three scenarios.

In scenario (a) (random binding), we find that over the range [*r*_1_, *r*_2_] the steady-state value of [*I*] is well approximated by a linear function of [*I*]*_T_*: 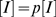 *_T_* 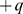. The goodness of fit as measured by *R* ^2^ was greater than 0.95 for 9,987 of the 10,000 iterations. For the 13 cases where *R* ^2^ was less than 0.95, we repeated the simulations with [*I*]*_T_* in the range [10*r*_1_, 10*r*_2_]. *R* ^2^ exceeded 0.98 in all 13 cases. For each choice of parameters, the fractional change in the steady-state value of [*I*] with respect to [*I*]*_T_* was calculated as follows:
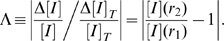
(M1)Thus, 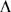 measures the percent change in the steady-state value of [*I*] as a result of doubling [*I*]*_T_*. (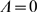 corresponds to perfect robustness of [*I*] with respect to [*I*]*_T_*.) We find that in over 95% of the simulations, 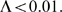 In over 99.7% of the simulations 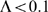. For all cases where 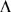 was found to exceed 0.1, we repeated the simulations with [*I*]*_T_* in the range [10*r*_1_, 10*r*_2_]. In all cases, the approximate linear dependence of [*I*] on [*I*]*_T_* was maintained, and 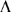 was now less than 0.1. In 24 out of 25 cases 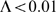 We therefore conclude that in a large range of [*I*]*_T_* values, the steady-state value of [*I*] is approximately robust with respect to [*I*]*_T_*.

Next, we focused on the case where the ternary complex has much more kinase than phosphatase activity 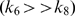 To study the limiting value of [*I*] as [*I*]*_T_* grows large, we performed 1,000 simulations as in scenario (a) above, but with 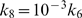. For each simulation, we evaluated the mean deviation of the steady-state value of [*I*] from the value *c*, as predicted by the leading term in (33). The deviation was calculated by the formula
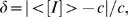
(M2)where <[*I*]> is the mean of the steady-state value of [*I*] over the range [*r*_1_,*r*_2_] of [*I*]*_T_* values. We found that in 983 out of 1000 simulations *δ* was less than 0.01. For the 17 cases where *δ* exceeded 0.01, we repeated the simulations with [*I*]*_T_* in the range [10*r*_1_, 10*r*_2_]. In all 17 cases *δ* was now less than 0.01. We therefore conclude that, in a large range of [*I*]*_T_* values, [*I*] is approximately equal to *c*, provided that the ternary complex has much more kinase than phosphatase activity.

We note that repeating the simulations of scenario (a), but with the ternary complex having more phosphatase than kinase activity 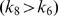, causes the robustness of [*I*] to be lost.

In scenario (b) (ordered binding with *E* binding *I_p_* first and then *EI_p_* binding *I*), we find that over the range [*r*_1_, *r*_2_], [*I*] is approximately a linear function of [*I*]*_T_*. In all 10,000 cases, *R* ^2^ exceeded 0.95, and in all cases 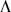 was less than 0.012. We therefore conclude that approximate robustness obtains in scenario (b).

In scenario (c) (ordered binding with *E* binding *I* first and then *EI* binding *I_p_*), we find that over the range [*r*_1_, *r*_2_] the steady-state value of [*I*] is a linear function of [*I*]*_T_* to very good approximation: In every case, *R* ^2^ was greater than 0.998. In all cases, 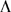 was found to be in the range [0.69, 1.58], and in 9,998 of the 10,000 cases, 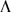 was found to be in the range [0.9, 0.1]. We therefore conclude that in case (c) robustness of the steady-state value of [*I*] with respect to [*I*]*_T_* is lost. Moreover, the fact that in the vast majority of cases 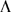 was approximately equal to 1 indicates that [*I*] is roughly proportional to [*I*]*_T_*.

In summary, we conclude that over the range of parameters tested, robustness of [*I*] requires that the ternary complex *EI_p_I* be assembled either in a random fashion or sequentially, with *E* binding *I_p_* first and then *EI_p_* binding *I*, and that the ternary complex's kinase activity exceed its phosphatase activity. This is summarized in Table 1.

**Table 1:** Robustness as a function of the mode of *EI_p_I* formation.

Mode of *EI_p_I* Formation	Robustness of [*I*]
Random order	Yes
Ordered, with *I_p_* binding *E* and *EI_p_* binding *I*	Yes
Ordered, with *I* binding *E* and *EI* binding *I_p_*	No

In all cases, the ternary complex is assumed to have more kinase than phosphatase activity, 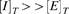, and 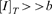.
